# Unique Clinically Relevant Prognostic Indicators After TIPS Placement in Cirrhosis Patients with Pre-Existing Kidney Disease

**DOI:** 10.3390/jcm15020414

**Published:** 2026-01-06

**Authors:** Rajesh Sasidharan, Cyriac Abby Philips, Akhil Baby, Tharun Tom Oommen, Arif Hussain Theruvath, Aryalakshmi Sreemohan, Ambily Baby, Rizwan Ahamed, Ajit Tharakan, Philip Augustine

**Affiliations:** 1 Department of Interventional Radiology, Center of Excellence in Gastrointestinal Sciences, Rajagiri Hospital, Aluva 683112, India; 2 Department of Clinical and Translational Hepatology, The Liver Institute, Center of Excellence in Gastrointestinal Sciences, Rajagiri Hospital, Aluva 683112, India; 3 The Division of Clinical Research, The Liver Institute, Center of Excellence in Gastrointestinal Sciences, Rajagiri Hospital, Aluva 683112, India; 4 Department of Gastroenterology and Advanced GI Endoscopy, Center of Excellence in Gastrointestinal Sciences, Rajagiri Hospital, Aluva 683112, India

**Keywords:** decompensated cirrhosis, HRS, ALD, MASLD, sepsis

## Abstract

**Background**: Transjugular intrahepatic portosystemic shunt (TIPSS) outcomes in patients with moderate-to-severe pre-existing kidney disease (PKD, stages G3a–G4) remain poorly characterized. This study aimed to identify potential predictors of mortality specifically in patients with an eGFR 15–59 mL/min/1.73 m^2^. **Methods**: We retrospectively analyzed 68 cirrhosis patients with PKD (eGFR < 60 mL/min/1.73 m^2^) undergoing a TIPSS between April 2021 and April 2024. Clinical outcomes, renal function changes, and 12-month survival were assessed. Statistical analyses included paired *t*-tests with false discovery rate adjustment and Kaplan–Meier survival analysis to identify potential predictors of mortality. **Results**: The cohort (mean age 61.0 ± 8.3 years, 83.8% male, 79.4% with PKD G3a–G3b) showed modest improvement in renal function (creatinine 1.93 to 1.75 mg/dL, *p* = 0.031), though this biochemical change did not predict survival. Overall mortality was 36.8% (95% CI: 25.4–49.5%) at mean follow-up of 6.7 months. Traditional severity scores (MELD, Child–Turcotte–Pugh) showed no significant association with survival (*p* > 0.05 for all comparisons). In exploratory analyses, mortality was significantly higher in patients with the following: (1) uncontrolled diabetes before a TIPSS (55.2% vs. 25.9%; RR 2.35, 95% CI: 1.08–5.15, *p* = 0.032); (2) post-TIPSS infection (70.0% vs. 31.0%; HR 5.44, 95% CI: 1.54–19.23, *p* = 0.009); and (3) post-procedural cardiac events (85.7% vs. 31.1%; *p* = 0.005). These associations persisted after false-discovery rate adjustment but require prospective validation given the modest sample size and wide confidence intervals. **Conclusions**: In this exploratory single-center study of patients with moderate PKD undergoing a TIPSS, we observed associations between mortality and pre-TIPSS poorly controlled diabetes, infections, and cardiac events. These hypothesis-generating findings suggest potential areas for future research. Prospective multi-center studies are needed to validate these associations and determine whether interventions targeting these factors improve outcomes.

## 1. Introduction

The transjugular intrahepatic portosystemic shunt (TIPSS) is a well-established, minimally invasive radiological procedure that has become a cornerstone therapy for managing severe complications of portal hypertension. By creating a low-resistance channel between the portal vein and the hepatic vein, a TIPSS effectively decompresses the portal venous system. This intervention is a primary treatment for refractory ascites (RA) and the prevention of recurrent variceal bleeding (AVB), with expanding indications that include hepatorenal syndrome (HRS) and hepatic hydrothorax [1]. Despite its proven efficacy, the profound hemodynamic and metabolic alterations induced by the shunt are associated with significant risks, most notably hepatic encephalopathy (HE) and, in poorly selected candidates, precipitous worsening of hepatic or cardiac failure [2]. Consequently, meticulous patient selection remains the single most critical determinant of procedural success and post-procedural survival.

The management of portal hypertension is significantly complicated by the presence of pre-existing kidney disease (PKD). Pre-existing kidney disease is a common and severe comorbidity in patients with cirrhosis, with a multifactorial pathogenesis that includes structural, organic renal disease (e.g., diabetic nephropathy, hypertensive nephrosclerosis) as well as functional, hemodynamically driven renal impairment (i.e., HRS). The presence of PKD is viewed by many clinicians as a relative contraindication to TIPSS placement, a caution supported by significant physiological concerns [3]. First, the kidney is responsible for a substantial portion of extrahepatic ammonia excretion; pre-existing renal impairment thus severely compromises ammonia homeostasis and synergistically amplifies the risk of post-TIPSS HE. Second, the presence of PKD is an independent risk factor for mortality in patients with cirrhosis, both on the liver transplant waitlist and following transplantation [4]. Finally, these patients are exceptionally vulnerable to the acute, massive increase in right atrial pressure and cardiac preload that immediately follows TIPSS creation [5]. The existing medical literature on TIPSS use in the PKD population is characterized by sparse, often conflicting data derived from small cohorts or large, non-granular databases. This has created a clinical evidence vacuum, making risk–benefit calculations highly uncertain.

The management of portal hypertension in patients with PKD represents a critical unmet clinical need in contemporary hepatology. While TIPSS placement has become standard care for RA and AVB, patients with PKD are systematically underrepresented in clinical trials. This exclusion creates an evidence gap that directly impacts clinical decision-making. The pathophysiological complexity of concurrent renal dysfunction presents unique challenges: these patients experience competing hemodynamic demands where TIPSS-induced changes in cardiac preload may overwhelm already compromised renal autoregulation, while paradoxically, the reduction in portal pressure might improve renal perfusion through decreased intra-abdominal pressure and improved systemic hemodynamics. Furthermore, PKD remains a ‘grey area’ for TIPSS placement, beckoning dedicated studies to establish risk stratification models. Our study directly addresses this knowledge gap by providing the largest cohort analysis to date of TIPSS outcomes specifically in PKD patients, offering clinically actionable insights for this challenging population.

Additionally, the decision to proceed with a TIPSS in PKD patients involves navigating competing risks that are poorly quantified in current literature. On one hand, these patients face accelerated mortality from RA and AVB, with studies showing 6-month mortality rates exceeding 50% with medical management alone. Conversely, TIPSS placement in PKD patients carries theoretical risks of precipitating cardiac decompensation from increased preload, worsening hepatic encephalopathy due to impaired renal ammonia clearance, and potentially accelerating progression to dialysis. Our systematic review of PubMed, EMBASE, and Cochrane databases (2000–2024) identified only seven studies specifically examining TIPSS outcomes in PKD patients, with the largest previous cohort containing merely 31 patients. This paucity of evidence has led to substantial practice variation, with some centers considering PKD a relative contraindication while others proceed with careful patient selection. Our study provides the largest cohort analysis to date, offering insightful outcome data that can inform shared decision-making in this complex clinical scenario.

Current medical literature consists of either small studies with granular data but low statistical power, or large administrative database studies that have power but lack the necessary clinical, procedural, and laboratory objectivity to capture critical post-procedural variables, laboratory dynamics, or specific complications to identify specific risk factors that drive clinical outcomes after a TIPSS. The clinical imperative for studying TIPSS outcomes specifically in patients PKD stems from the convergence of epidemiological burden and physiological vulnerability. The most significant lacuna is the lack of data on actionable predictors of survival. Knowing that “PKD” is a risk factor on TIPSS outcomes is not clinically actionable. Hence it is imperative to understand why these patients fare poorly. We do not have clear, validated, modifiable targets for intervention that clinicians can address to improve outcomes. Therefore, the present study was conducted not merely to report outcomes, but to perform a more incisive analysis to identify novel, non-traditional predictors of mortality in a large cohort of cirrhosis patients with PKD undergoing a TIPSS. The specific objectives of this study were as follows: (1) to describe clinical outcomes including renal function changes, hepatic encephalopathy, hospital readmissions, and transplant-free survival in cirrhosis patients with moderate-to-severe PKD (eGFR < 60 mL/min/1.73 m^2^) undergoing a TIPSS; (2) to identify clinical, procedural, and metabolic factors associated with mortality in this population; and (3) to generate hypotheses for potentially modifiable risk factors that could inform future prospective studies and clinical practice.

## 2. Materials and Methods

### 2.1. Patients

We retrieved detailed data from cirrhosis patients with PKD undergoing TIPSS placement for various indications from April 2021 to April 2024.

Inclusion criteria included the following:

(1) Age ≥ 18 years; (2) established diagnosis of cirrhosis confirmed by imaging, histology, or clinical/laboratory criteria; (3) pre-existing kidney disease defined as eGFR < 60 mL/min/1.73 m^2^ [Kidney Disease: Improving Global Outcomes (KDIGO) 2012 Clinical Practice Guidelines category G3a or worse] documented on at least two measurements separated by ≥3 months prior to TIPSS to ensure chronicity; and (4) TIPSS placement for portal hypertension complications.

Exclusion criteria included the following:

(1) Acute kidney injury (AKI) without established PKD, defined as patients meeting KDIGO AKI criteria without prior documentation of reduced eGFR; (2) non-cirrhotic portal hypertension; (4) prior TIPSS placement or revision; (5) active malignancy; and (6) renal replacement therapy (dialysis) at time of TIPSS.

The eGFR was calculated using the PKD-EPI 2021 equation without race coefficient, as recommended by current guidelines. Patients with mixed etiologies of kidney disease (e.g., diabetic nephropathy and hepatorenal physiology) were included, as this reflected real-world practice where pure etiologies are uncommon. The presence of structural renal disease on imaging (chronic changes such as cortical thinning, increased echogenicity, or reduced kidney size) was noted. We did not attempt to distinguish functional from structural components of renal dysfunction, as this distinction is often clinically impossible without renal biopsy. We analyzed 12-month clinical outcomes inclusive of changes to liver disease severity and renal function, portal hypertension events, hospitalization, and transplant-free survival in cirrhosis patients with PKD. All participants (or their immediate family members) provided informed consent for the treatments and for the use of their deidentified reports for future research. This study was approved by the Institutional Ethics and Review Board of Rajagiri Hospital and was performed in conformance with the Helsinki declaration of 1975 and its pertinent revisions.

### 2.2. Definitions

Glycemic status was classified based on measurements obtained within 3 months prior to TIPSS, with controlled diabetes defined as HbA1c < 7.5%, while uncontrolled diabetes was defined as HbA1c ≥ 7.5%. Post-procedural glycemic deterioration was defined as any of the following: worsening glycemia (increase in HbA1c or mean fasting glucose values compared to baseline) within 1 month of the procedure, new-onset diabetes diagnosed by HbA1c criteria, or development of diabetes-related symptoms (polyuria, polydipsia, unexplained weight loss, or diabetic ketoacidosis) within 3 months following TIPSS. All glucose measurements were obtained from point-of-care capillary samples or venous samples during routine laboratory draws. Post-TIPSS infection was defined as any culture-positive or clinically diagnosed infection requiring antibiotic therapy in-hospital after TIPSS or within 30 days after discharge. Cardiac events included symptomatic arrhythmia, cardiac-related dyspnea, or heart failure requiring hospital care at any point during follow-up. Intraprocedural hypotension was defined as systolic blood pressure < 90 mmHg or mean arterial pressure < 65 mmHg requiring intervention.

### 2.3. Pre-TIPSS Standard Cardiac Evaluation

All patients underwent standardized cardiac evaluation before TIPSS placement as follows: resting transthoracic echocardiography (TTE) within 30 days before procedure to assess left ventricular ejection fraction (LVEF), right ventricular systolic pressure (RVSP), evidence of cirrhotic cardiomyopathy (diastolic dysfunction, E/A ratio), and chamber dimensions. Additional screening tests included electrocardiography (ECG) within 24 h of procedure and cardiology consultation for patients with known coronary artery disease, abnormal LVEF < 50%, and RVSP > 40 mmHg. Notably, functional stress testing or biomarkers of acute coronary disease or heart failure were not routinely performed due to limitations in exercise capacity in this population and lack of standardized protocolized recommendations.

### 2.4. Statistical Methods

Statistical analysis was performed using MedCalc^®^ version 23.0.5 (Ostend, Belgium). Continuous variables were analyzed using descriptive statistics with 95% confidence intervals. For non-normally distributed variables, logarithmic transformation was applied with back-transformation for clinical interpretation. Pre- and post-procedural comparisons used paired samples *t*-tests with Kolmogorov–Smirnov testing for normality. To address multiple comparisons, we applied the Benjamini–Hochberg procedure for false discovery rate (FDR) control with FDR set at 0.10 for primary outcomes. Both uncorrected *p*-values and FDR-adjusted *q*-values are reported. Categorical variables were analyzed using chi-squared tests. Time-to-event analyses employed Kaplan–Meier curves with log-rank tests. Hazard ratios with 95% confidence intervals were calculated using Cox proportional hazards models. Multivariable models were adjusted for age, sex, MELD score, ACLF Grade, and kidney disease stage. E-values were calculated to assess sensitivity to unmeasured confounding. Statistical significance was set at *p* < 0.05, with two-tailed tests throughout.

## 3. Results

### 3.1. Study Population and Baseline Characteristics

#### Patient Demographics and Clinical Presentation

A total of 68 patients with PKD who underwent a TIPSS placement for complications related to portal hypertension were included in this analysis. The cohort had a mean age of 61.0 ± 8.3 years (range: 45–78 years), with 57 patients (83.8%) being male and 11 (16.2%) females. The etiology of cirrhosis was evenly distributed between alcohol-related liver disease (ALD; n = 34, 50.0%) and metabolic dysfunction-associated steatohepatitis (MASH; n = 33, 48.5%). At the time of the procedure, 37 patients (54.4%) had no acute-on-chronic liver failure (ACLF Grade 0), while 17 (25.0%) had ACLF Grade 1 and 14 (20.6%) had ACLF Grade 2.

### 3.2. Kidney Function Classification

**Figure 1:**
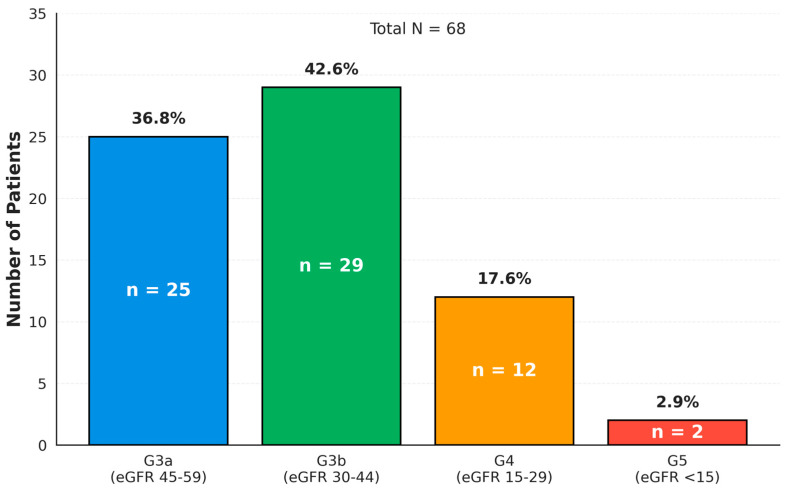
Distribution of pre-existing kidney disease (PKD) stages among 68 patients with cirrhosis undergoing transjugular intrahepatic portosystemic shunt (TIPSS) placement. This bar chart illustrates the stratification of the study population by PKD severity based on estimated glomerular filtration rate (eGFR) categories according to KDIGO (Kidney Disease: Improving Global Outcomes) classification. The *x*-axis displays PKD stages with corresponding eGFR ranges in mL/min/1.73 m^2^. The *y*-axis represents the number of patients in each category.

The mean baseline eGFR was 38.5 ± 11.2 mL/min/1.73 m^2^ (median: 41.5, IQR: 40.0–43.6). All patients had significant renal dysfunction at baseline, with predominantly G3b PKD (42.6%) (Figure 1). The renal disease stages were distributed as follows:

- G3a (eGFR 45–59 mL/min/1.73 m^2^): 25 patients (36.8%);
- G3b (eGFR 30–44 mL/min/1.73 m^2^): 29 patients (42.6%);
- G4 (eGFR 15–29 mL/min/1.73 m^2^): 12 patients (17.6%);
- G5 (eGFR < 15 mL/min/1.73 m^2^): 2 patients (2.9%).

### 3.3. Comorbidities

Diabetes mellitus was present in 57 patients (83.8%), with 27 (48.2%) having controlled diabetes and 29 (51.8%) having uncontrolled diabetes at baseline. Hypertension was documented in 21 patients (30.9%), while hypothyroidism was rare (n = 2, 3.0%). On imaging, 22 patients (32.4%) showed chronic renal disease changes in the kidneys.

### 3.4. TIPSS Procedure Characteristics

The majority of procedures (n = 56, 82.4%) were performed electively, with 12 (17.6%) performed as emergency interventions. The primary indications for TIPSS placement included the following: refractory ascites: 37 patients (54.4%); acute variceal bleeding: 17 patients (25.0%); and recurrent ascites: 14 patients (20.6%). The mean preprocedural fasting time was 12.7 h (geometric mean, range: 6–48 h), with a median total procedural time of 73 min (geometric mean: 73.9, range: 45–180 min).

### 3.5. Baseline Laboratory Parameters and Severity Scores

Baseline laboratory values demonstrated significant hepatic and renal dysfunction (Table 1). The mean baseline Child–Turcotte–Pugh (CTP) score was 8.4 ± 1.5 (median: 8.5, range: 5–12), indicating predominantly Class B cirrhosis. The mean baseline MELD 3.0 score was 22.4 ± 5.8 (median: 22.8, range: 10.2–35.4). The mean CLIF-C AD score was 59.4 ± 8.6, with a mean CLIF-C organ failure score of 6.7.

#### TIPSS Technical Parameters

NITI stents were predominantly used (69.1%), followed by Hanaro (20.6%), BARD (8.8%), and Epic (1.5%) stents, with the use of 10 mm diameter stents predominating (82.4%) over 8 mm (17.6%) sizes. Complete details of hemodynamic assessments were available in 56 patients. Portal pressure measurements demonstrated a mean pre-TIPSS gradient of 18.3 ± 4.2 mmHg reduced to a post-TIPSS gradient of 7.8 ± 2.1 mmHg, representing a 57.4 ± 12.8% reduction with 95.6% achieving target gradient < 12 mmHg.

### 3.6. Immediate Post-Procedural Changes

#### 3.6.1. Laboratory Parameters

Total bilirubin increased significantly from 1.48 to 2.28 mg/dL (geometric mean ratio: 1.54, 95% CI: 1.36–1.75, *p* < 0.001). The coagulation parameter INR increased from 1.56 to 1.82 (95% CI: 1.12–1.22, *p* < 0.001). Nonetheless, serum creatinine showed a modest but significant decrease from 1.93 to 1.75 mg/dL (95% CI: 0.83–0.99, *p* = 0.031). Total leukocyte count increased from 6.2 to 8.2 × 10^3^/μL (geometric mean ratio: 1.32, 95% CI: 1.18–1.48, *p* < 0.001). Hemoglobin remained stable (9.1 to 9.2 g/dL, *p* = NS) as did the platelet count (116.8 to 119.7 × 10^3^/μL, *p* = NS). Interestingly, serum sodium increased from 133.4 to 136.5 mEq/L (*p* < 0.001). The CTP score increased modestly from 8.27 to 8.74 (geometric mean ratio: 1.06, 95% CI: 1.01–1.10, *p* = 0.016), while the MELD 3.0 score remained unchanged (21.6 to 21.8, *p* = 0.74) (Table 2).

#### 3.6.2. Procedural Complications and Immediate Outcomes

Intraprocedural hypotension requiring intervention occurred in eleven patients (16.2%), of whom seven (63.6%) required vasopressor support (norepinephrine in five patients, vasopressin in two patients). The choice of intravenous fluids reflected hemodynamic severity: patients receiving balanced crystalloids plus colloids (n = 8) had higher baseline MELD scores (26.3 vs. 21.8, *p* = 0.018) and more frequent vasopressor requirements (62.5% vs. 8.3%, *p* < 0.001) compared to those receiving crystalloids alone. Thus, the association between fluid type and mortality (*p* = 0.041) likely represents confounding by illness severity rather than a causal effect of fluid choice. Ischemic hepatitis developed in 16 patients (23.5%) post-procedure.

### 3.7. Hospital Course and Clinical Outcomes

#### 3.7.1. Length of Stay, Post-Procedural Complications, and In-Hospital Mortality

The mean ICU stay was 1.1 ± 1.6 days with many patients not requiring ICU admission. The mean total hospital stay was 6.3 days (median: 7.0 days, range: 2–22 days). Eight patients (11.8%) died during the index hospitalization. The causes of in-hospital death were predominantly sepsis in four patients (50.0%) followed by progressive liver failure and multiple organ failure in three patients (37.5%) while one patient (12.5%) developed cardiac failure.

Post-TIPSS infections developed in 10 patients (14.7%). Hepatic encephalopathy occurred in 17 patients (25.0%) at any time during follow-up, despite only 9 patients (13.2%) having a history of prior HE before the procedure. Significant cardiac events (defined as an event requiring emergency visit or admission—symptomatic arrythmias, dyspnea of cardiac origin, or clinically-relevant cardiac failure) post-TIPSS—occurred in seven patients (10.3%).

#### 3.7.2. 30-Day and 90-Day Outcomes

Fourteen patients (20.6%) required readmission within 30 days. The predominant reasons for 30-day readmission included hepatic encephalopathy: five patients (35.7%), sepsis: three patients (21.4%), and symptomatic ascites: two patients (14.3%). Ten patients (14.7%) required readmission beyond 90 days. The predominant reasons included dyselectrolytemia: five patients (45.5%), hepatic encephalopathy: two patients (18.2%) and sepsis: two patients (18.2%) (Table 3).

#### 3.7.3. Overall Survival and Predictors of Mortality

During a mean follow-up period of 6.7 months, 25 of 68 patients (36.8%) died. Patients were censored at the time of last follow-up, death, or 12 months, whichever occurred first. Eight deaths occurred during index hospitalization (median time: 1 month), while seventeen occurred post-discharge (median time: 5 months).

Among patients with diabetes, those with uncontrolled diabetes had significantly higher mortality compared to those with controlled diabetes (55.2% vs. 25.9%, *p* = 0.028). The relative risk of death for uncontrolled diabetes was 2.13 (95% CI: 1.04–4.36, *p* = 0.039), with an odds ratio of 3.52 (95% CI: 1.14–10.88). Similarly, uncontrolled post-procedural glycemia was associated with increased mortality across the entire cohort (48.7% vs. 20.7%, *p* = 0.019), with a relative risk of 2.35 (95% CI: 1.08–5.15, *p* = 0.032) and an odds ratio of 3.64 (95% CI: 1.22–10.90). Patients who developed post-TIPSS infections had dramatically higher mortality (70.0% vs. 31.0%, *p* = 0.019). Survival analyses revealed a hazard ratio of 5.44 (95% CI: 1.54–19.23, *p* = 0.009).

**Figure 2:**
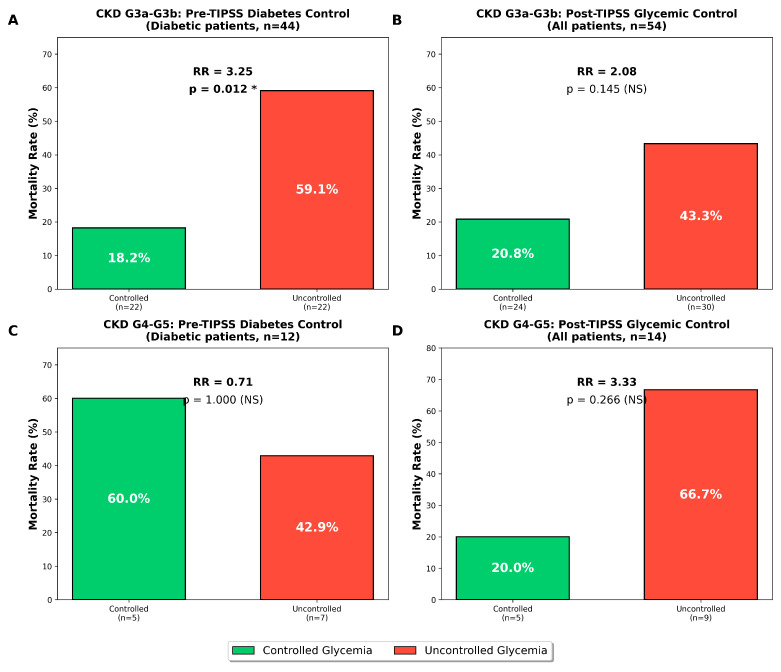
Stratified analysis of glycemic control effects on mortality by PKD stage in TIPSS patients—mortality rates for controlled (green) versus uncontrolled (red) glycemia. (**A**): Pre-TIPSS diabetes control in diabetic patients with moderate PKD (n = 44). Mortality was 18.2% in controlled (n = 22) versus 59.1% in uncontrolled patients (n = 22). Relative risk = 3.25, *p* = 0.012. (**B**): Post-TIPSS glycemic control in all patients with moderate PKD (n = 54), regardless of diabetes history. Mortality was 20.8% in controlled (n = 24) versus 43.3% in uncontrolled patients (n = 30). Relative risk = 2.08, *p* = 0.145 (NS). (**C**): Pre-TIPSS diabetes control in diabetic patients with advanced PKD (n = 12). Mortality was 60.0% in controlled (n = 5) versus 42.9% in uncontrolled patients (n = 7), which was not significant. Panel (**D**): Post-TIPSS glycemic control in all patients with advanced PKD (n = 14). Mortality was 20.0% in controlled (n = 5) versus 66.7% in uncontrolled patients (n = 9). Relative risk = 3.33, *p* = 0.266 (NS). Fisher’s exact test was used for all comparisons due to small sample sizes. Relative risks (RRs) were calculated as the ratio of mortality rates between groups. Two tailed *p*-values < 0.05 were considered statistically significant (indicated by asterisk). NS = not significant.

When stratified by PKD severity (Figure 2), among PKD G3a–G3b (n = 54, 18 deaths), infection remained significantly associated with mortality (HR 2.86, 95% CI: 1.02–8.05, *p* = 0.046). However, it revealed differential impacts of glycemic control on mortality. In patients with moderate PKD (G3a–G3b, n = 54), uncontrolled pre-TIPSS diabetes was associated with significantly higher mortality compared to controlled diabetes (59.1% vs. 18.2%, *p* = 0.012; RR 3.25, 95% CI: 1.25–8.42). Post-TIPSS uncontrolled glycemia in this group showed a similar though non-significant harmful trend (43.3% vs. 20.8%, *p* = 0.145; RR 2.08, 95% CI: 0.86–5.02). In contrast, patients with advanced PKD (G4–G5, n = 14) demonstrated inconsistent patterns: pre-TIPSS diabetes control showed no significant association with mortality (42.9% vs. 60.0%, *p* = 1.00), while post-TIPSS uncontrolled glycemia showed a non-significant harmful trend (66.7% vs. 20.0%, *p* = 0.266; RR 3.33, 95% CI: 0.54–20.43). These findings suggest that glycemic control is particularly important in moderate PKD patients undergoing a TIPSS, where uncontrolled diabetes more than triples mortality risk. The limited sample size in the advanced PKD group (n = 14) and high baseline mortality (50%) may have obscured glycemic effects in this population.

Post-TIPSS cardiac events (symptomatic heart-specific events requiring hospitalization) were strongly associated with mortality (85.7% vs. 31.1%, *p* = 0.005). Patients experiencing intraprocedural hypotension had higher in-hospital mortality (36.4% vs. 7.0%, *p* = 0.006) and worse overall survival (Table 4).

The type of IV fluid used during the procedure was associated with in-hospital mortality (*p* = 0.041), with patients given balanced crystalloids plus colloids, but not either alone, having the highest mortality rate. The Child–Pugh or MELD3 score, or presence of prior HE or post-TIPSS HE episodes did not significantly affect clinical outcomes. However, ACLF Grade was significantly associated with overall mortality [Grade 2, HR 2.34 (95% CI 1.01–5.42), *p* = 0.047). Baseline MELD3 ≥ 25, higher age > 65 years, indication for TIPSS placement (variceal bleeding vs. ascites), categories of PKD, and procedural timing (elective vs. emergency) did not significantly impact in-hospital or overall mortality.

In multivariable Cox regression models adjusting for age, sex, MELD score, ACLF Grade, PKD stage, and baseline cardiovascular disease, post-procedural infection (adjusted HR 3.23, 95% CI: 1.25–8.37, *p* = 0.016) and cardiac events (adjusted HR 7.74, 95% CI: 2.50–23.97, *p* < 0.001) remained significantly associated with mortality. Hyperglycemia did not show statistically significant association. E-value analysis indicated that unmeasured confounders would need risk ratios of 5.84 (lower CI: 2.00) for infection and 7.70 (lower CI: 2.66) for cardiac events to explain away these associations, suggesting moderate robustness to unmeasured confounding. The non-significant glycemia finding warrants cautious interpretation and may reflect limitations inherent to retrospective data collection, timing of glucose measurements, or survival bias, highlighting the need for prospective validation.

**Figure 3:**
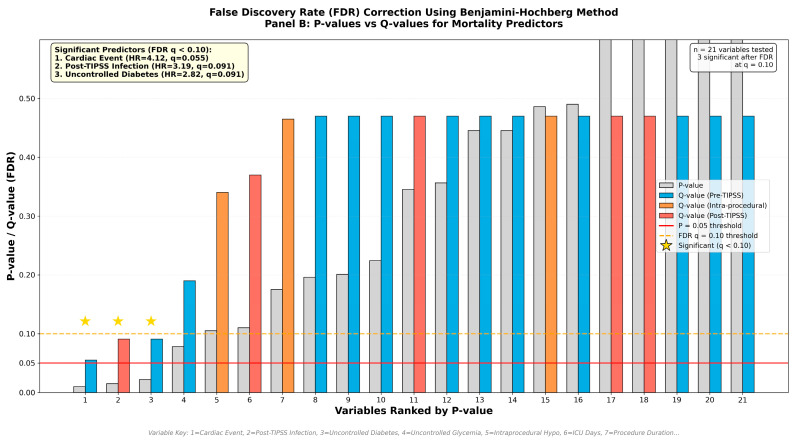
False Discovery Rate correction of mortality predictors—*p*-values (gray) and Q-values (colored by category: blue = pre-TIPSS, orange = intra-procedural, red = post-TIPSS) for 21 variables ranked by significance. Stars indicate significant predictors after Benjamini–Hochberg correction (Q < 0.10): cardiac events (represented by variable 1 on *x*-axis), infection (represented by variable 2), and uncontrolled diabetes pre-TIPSS (represented by variable 3). Horizontal lines show *p* = 0.05 and FDR Q = 0.10 thresholds. Other variables of interest that were not significant include baseline MELD score (9), baseline PKD stage (10), age (12), presence of ACLF (13), Child–Pugh class C (14), and encephalopathy post-TIPSS (18).

To address multiple testing concerns, we applied the Benjamini–Hochberg false discovery rate (FDR) correction to all potential mortality predictors (Figure 3). Among 21 variables tested across pre-TIPSS (n = 13), intra-procedural (n = 2), and post-TIPSS (n = 6) categories, three predictors remained significant after FDR correction at *q* < 0.10. Post-TIPSS cardiac events demonstrated the strongest association with mortality (HR 4.12, 95% CI: 1.64–10.36; *p* = 0.003, *q* = 0.055), followed by post-TIPSS infection (HR 3.19, 95% CI: 1.33–7.66; *p* = 0.009, *q* = 0.091) and pre-TIPSS uncontrolled diabetes (HR 2.82, 95% CI: 1.24–6.38; *p* = 0.013, *q* = 0.091). While post-TIPSS uncontrolled glycemia and intraprocedural hypotension showed elevated hazard ratios, they did not survive multiple comparison correction. The persistence of cardiac events, infection, and uncontrolled diabetes as significant predictors after rigorous FDR adjustment strengthens the evidence for their role as key determinants of mortality in PKD patients undergoing a TIPSS.

**Figure 4:**
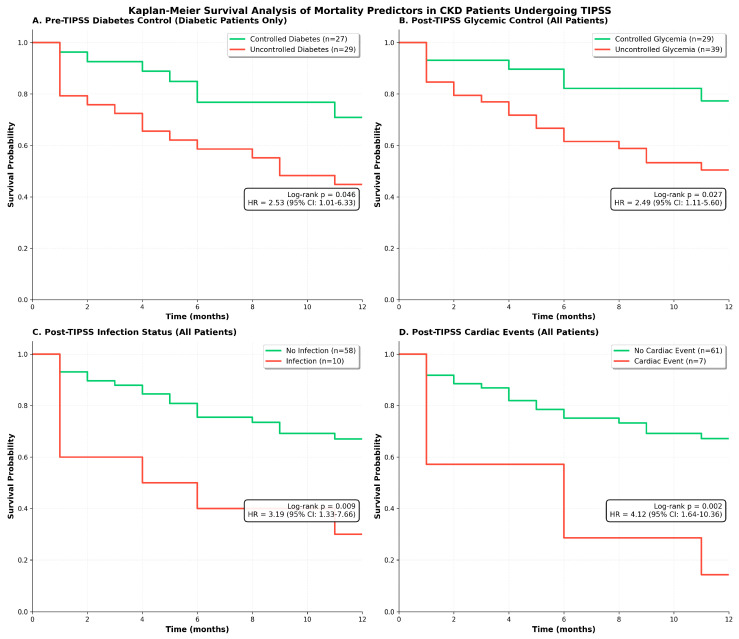
Four key predictors of post-TIPSS mortality are shown over 12 months of follow-up. (**A**) Pre-TIPSS diabetes control among cirrhosis with diabetes (n = 56) demonstrates significantly worse survival in patients with uncontrolled diabetes (red) versus controlled diabetes (green) (HR = 2.53, 95% CI: 1.01–6.33, log-rank *p* = 0.046). (**B**) Post-TIPSS glycemic control in all patients (n = 68) shows uncontrolled glycemia (red) associated with increased mortality compared to controlled glycemia (green) (HR = 2.49, 95% CI: 1.11–5.60, *p* = 0.027). (**C**) Post-TIPSS infection status reveals markedly reduced survival in patients developing infections (red, n = 10) versus those without infections (green, n = 58) (HR = 3.19, 95% CI: 1.33–7.66, *p* = 0.009), with early curve separation indicating rapid clinical deterioration. (**D**) Post-TIPSS cardiac events demonstrate the strongest association with mortality, with cardiac events (red, n = 7) resulting in 86% mortality compared to 31% in patients without cardiac events (green, n = 61) (HR = 4.12, 95% CI: 1.64–10.36, *p* = 0.002). Hazard ratios were calculated using Cox proportional hazards regression, and survival differences were assessed using the log-rank test.

Kaplan–Meier survival analysis (Figure 4) revealed four significant predictors of mortality in PKD patients undergoing a TIPSS. Among diabetic patients (n = 57), uncontrolled pre-TIPSS diabetes was associated with significantly worse survival compared to controlled diabetes (45% vs. 74% survival, log-rank *p* = 0.046), with 16 deaths among 29 uncontrolled versus 7 deaths among 27 controlled diabetic patients. Post-TIPSS glycemic control demonstrated similar prognostic importance across all patients (n = 68), with uncontrolled glycemia associated with markedly reduced survival compared to controlled glycemia (51% vs. 79%, log-rank *p* = 0.027). Post-procedural complications showed even stronger associations with mortality. Post-TIPSS infection, occurring in ten patients (14.7%), was associated with dramatic early mortality, with only 30% survival compared to 69% in non-infected patients (log-rank *p* = 0.009), suggesting rapid clinical deterioration following infectious complications. Most strikingly, post-TIPSS cardiac events, though occurring in only seven patients (10.3%), demonstrated the strongest association with mortality, with 86% mortality (6/7 patients) compared to 31% in those without cardiac events (log-rank *p* = 0.002). No significant difference in survival was observed based on the indication for TIPSS, pre- or post-TIPSS encephalopathy, as well as acute variceal bleeding vs. refractory or recurrent ascites.

Given the modest sample size (n = 68, 25 events), subgroup analyses should be interpreted with caution. Wide confidence intervals reflect substantial uncertainty, and some associations may be unstable due to small cell sizes. With only 25 deaths, multivariable models were limited by the events-per-variable constraint, and adjusted estimates should be considered exploratory.

### 3.8. Sensitivity Analysis by PKD Stage

Given the small number of patients with advanced PKD (G4: n = 12, G5: n = 2), we performed a sensitivity analysis combining G4 and G5 into an “advanced PKD” category. Even with this grouping, no significant association with mortality was observed (advanced PKD mortality: 50.0% vs. G3a–G3b: 33.3%, *p* = 0.35). However, post-hoc power analysis revealed only 20–30% power to detect a clinically meaningful difference with this sample size. A sample of approximately 150–200 patients would be required to adequately assess the impact of PKD stage on mortality, which requires prospective evaluation.

## 4. Discussion

Our cohort of 68 patients substantially exceeds most cited studies on TIPSS in PKD, including Lakhoo et al.’s 17-patient G4–G5 cohort [6]. The population had severe disease (mean MELD 22.4, CTP 8.4) comparable to Lakhoo’s high-risk cohort, with advanced renal dysfunction—63.1% having G3b or higher PKD, including 20.5% with G4/G5 disease—ensuring relevance to the most challenging patient subgroup. The near-even split between ALD and MASH reflects modern hepatology practice, with the high MASH prevalence underlying the exceptional 83.8% diabetes rate, a key comorbidity central to our unique findings.

Our analysis found statistically significant renal function improvements following TIPSS, with mean creatinine decreasing from 1.93 to 1.75 mg/dL, aligning with prior studies: Ponzo et al. reported 1.94 to 1.37 mg/dL in HRS-CKD patients [7], Anderson et al. noted improvement from >2.0 to 1.5 mg/dL [8], and Lakhoo et al. observed GFR improvement from 20.0 to 42.2 mL/min in G4–G5 patients [6]. However, this statistically significant reduction does not establish clinically meaningful nephroprotection in PKD patients—the modest improvement likely reflects enhanced renal perfusion from reduced abdominal pressure rather than true kidney injury reversal. Critically, this biochemical change did not translate to survival benefit, and without longitudinal data, we cannot assess the sustainability of even this modest effect.

The mechanism for this improvement was strongly suggested by another significant finding from our study: the simultaneous significant increase in mean serum sodium from 133.4 mEq/L to 136.5 mEq/L. Hyponatremia is a well-established surrogate for extreme neurohormonal activation and is a predictor of poor outcomes [9]. The concurrent, significant correction of both serum creatinine and serum sodium strongly implies that the primary benefit of TIPSS is hemodynamic. The procedure improves effective circulatory volume, which in turn attenuates the systemic vasodilation and compensatory renal vasoconstriction that defines hepatorenal physiology.

The critical clinical implication concerns the “hybrid” nature of PKD in cirrhosis. Clinicians often withhold TIPSS from patients with elevated creatinine and structural renal disease (e.g., diabetic nephropathy), fearing “fixed” dysfunction will yield only complications without renal benefit [10]. Our cohort—83.8% diabetic and 32.4% with chronic renal disease on imaging—represented precisely this ‘feared’ population yet still demonstrated significant creatinine improvement. This provides robust evidence that even established structural renal disease includes a reversible functional component driven by portal hypertension that a TIPSS successfully addresses. Therefore, elevated creatinine in PKD patients should not contraindicate a TIPSS; rather, it may indicate significant hepatorenal dysfunction warranting hemodynamic correction. Supporting this, renal disease categories did not predict worse clinical outcomes in our cohort.

The procedural complications and immediate outcomes reported in this study provide a crucial window into the hemodynamic fragility of this high-risk PKD population. The 16.2% incidence of intraprocedural hypotension was a clinically significant finding; these patients, often with cirrhotic cardiomyopathy and a pre-existing hyperdynamic circulation, are exceptionally vulnerable to the acute hemodynamic shifts of anesthesia and shunt creation. This event is not benign, as confirmed by our study’s own data linking it directly to a five-fold increase in in-hospital mortality (36.4% vs. 7.0%). This finding is strongly supported by broader surgical and procedural literature, which identifies perioperative hypotension as a powerful, independent predictor of early mortality [11]. Hospitalization for cardiac decompensation was observed in 20% of patients in the year after TIPSS insertion in a recent study [12]. In our study, the presence of clinically significant cardiac events was associated with worse outcomes on follow up.

The hemodynamic instability was reflected in varied fluid regimens: 42.6% received crystalloids alone, 33.8% balanced crystalloids, and the remainder colloids or combinations—highlighting ongoing debate about optimal fluids in critically ill renal patients. The significant association between combined fluid therapy (balanced crystalloids + colloids) and in-hospital mortality is crucial yet complex. Single-fluid-type recipients are typically hemodynamically stable, receiving maintenance or replacement therapy. Conversely, patients requiring fluid combinations are experiencing severe hemodynamic distress—likely refractory hypotension, which independently showed five-fold increased mortality. These cases represent therapy escalation: after crystalloid resuscitation fails, colloids are added for rapid, sustained intravascular expansion [13]. Thus, combination fluid use serves as a surrogate marker for the most critically ill patients already failing initial resuscitation and tracking toward poor outcomes.

Our 25% hepatic encephalopathy incidence was substantially lower than Lakhoo’s 47% in G4–G5 PKD patients [6] and the typical 30–50% in the post-TIPSS range, suggesting HE risk is not linearly related to declining GFR but involves a threshold effect. Our cohort was predominantly G3a (36.8%) and G3b (42.6%), with 79.4% having an eGFR > 30 mL/min/1.73 m^2^, contrasting with Lakhoo’s cohort that was entirely <30. This threshold concept is validated by Li et al.’s finding that GFR 30–60 was not predictive of post-TIPSS HE, while a GFR < 30 strongly predicted it (OR 3.56, *p* = 0.023) [10]. Our 25% HE rates in this G3-dominant cohort provides crucial clinical validation that a TIPSS in G3 PKD (eGFR 30–59) may not carry the prohibitive HE risks seen in G4/G5 patients, as the kidneys likely retain sufficient functional reserve for extrahepatic ammonia excretion.

Our 30-day readmission rate of 20.6% compares favorably to 21–27.81% in general TIPSS cohorts [14,15] and the 58% reported for high MELD patients [15], suggesting robust management despite our high-risk population (mean MELD 22.4). Readmissions were driven by hepatic encephalopathy (35.7%) and sepsis (21.4%), mirroring Khan’s findings (HE 36.43%) [15], reinforcing the need for post-discharge HE prophylaxis and infection surveillance. The 11.8% in-hospital mortality and 36.8% overall mortality at 6.7 months follow-up align with high-risk PKD literature—Li’s 13.7% in-hospital rate [10], Lakhoo’s 29% 90-day mortality in G4–G5 patients [6], and 32% 1-year mortality cited by Li and Sarin [10]—confirming poor prognosis in dual-organ impairment. While these mortality rates are not novel, our critical contribution identifies why these patients die, moving beyond static MELD/CTP scores to reveal novel, potentially modifiable death predictors.

Our study identified three potentially important mortality predictors not captured by standard models. First, our exploratory analysis suggested a potential association between uncontrolled diabetes and increased mortality risk—supported by Li et al.’s findings on “DM with chronic complications” [10], with hyperglycemia’s pro-inflammatory, pro-thrombotic effects impairing immune function in already-compromised hosts [16], suggesting consideration of perioperative glycemic control as a potential therapeutic strategy. Second, post-TIPSS infections proved catastrophic with 70% mortality versus 31% in infection-free patients, representing a potentially important predictor (HR double that of poor glycemic control), with sepsis causing 50% of in-hospital deaths [17,18]—reflecting “triple-immunocompromise” from cirrhosis, renal disease, and procedural stress, mandating adequate infection surveillance and questioning whether the standard single-dose prophylaxis suffices. Third, post-TIPSS cardiac events yielded 85.7% mortality, far exceeding the 46% one-year survival in general cohorts [19], with intraprocedural hypotension also potentially predicting mortality (36.4% vs. 7.0%), indicating that the standard pre-TIPSS cardiac clearance with resting echocardiography maybe insufficient—future protocols should consider dobutamine stress echocardiography, right heart catheterization, cardiac biomarkers, or cardiopulmonary testing to assess reserve capacity before subjecting these fragile ventricles to TIPSS’s acute preload challenge.

In summary, the “clinical triad” concept identifies three interconnected mortality drivers following a TIPSS—uncontrolled diabetes, infection, and cardiac events—that operate synergistically independent of traditional liver scores. Post-procedural hyperglycemia impairs immunity and promotes bacterial translocation, creating a substrate for infection, which triggers systemic inflammation that worsens insulin resistance while precipitating organ failure. Both hyperglycemia and infection-induced inflammation stress an already compromised cardiovascular system, where TIPSS-related preload increases, cytokine-mediated myocardial depression, and underlying cirrhotic cardiomyopathy combine to produce cardiac events with 85.7% mortality. This creates a self-perpetuating cycle: hyperglycemia predisposes to infection, infection worsens glycemic control and cardiac function, and cardiac dysfunction impairs metabolic homeostasis and immune response, explaining why mortality escalates dramatically with multiple components present and mandating simultaneous targeting of all three elements rather than isolated interventions.

Despite the novel findings, this analysis has several important limitations. The study was retrospective and from a single center, which introduces inherent selection bias and limits the external generalizability of the findings. While the cohort (N = 68) was one of the largest granular cohorts of its kind, it remains modest. The cohort’s demographic characteristics (83.8% male, 83.8% diabetes) reflect the etiological distribution (ALD 50%, MASLD 48.5%) and may limit generalizability to populations with different cirrhosis etiologies or sex distributions Additionally, the mean follow-up of 6.7 months was relatively short, which is appropriate for capturing the high-risk early post-procedural period but limits conclusions regarding 1-year or long-term survival. The kidney disease cohort was also heterogeneous, spanning G3a to G5, and the small number of patients with G4/G5 disease (n = 14) limits the ability to draw specific conclusions for the advanced renal disease population, where risks are likely highest. Our study was underpowered to detect mortality differences across PKD stages, particularly for advanced PKD (G4–G5). Despite multivariable adjustment, residual confounding was likely. We lacked data on several potentially important confounders including nutritional status, sarcopenia assessment, infection source and severity, intensity of post-TIPSS monitoring, and socioeconomic factors. The associations we report should be considered hypothesis-generating rather than causal. The high diabetes prevalence makes it difficult to disentangle diabetes-specific effects from PKD-specific effects; the observed associations may reflect diabetes-related complications rather than kidney disease per se. Our glycemic control definitions, while based on established clinical guidelines, could not be subjected to sensitivity analyses due to the heterogeneous nature of glucose data collection in our retrospective design, the limited sample size of diabetic patients and the absence of standardized timing for glucose measurements relative to meals, medications, and procedural stress. These constraints reflect real-world clinical practice but preclude exploration of alternative glycemic thresholds. Future studies with more diverse etiological distributions would help clarify this relationship. The lack of association between PKD severity and outcomes may reflect a Type II error rather than true equivalence. Larger multi-center studies are needed to definitively assess whether PKD stage independently affects post-TIPSS mortality. Finally, key predictive variables were reported without precise objective quantification based on operational definitions and the analysis lacked a concurrent non-PKD control group, necessitating comparisons to published historical and external cohorts, which is a weaker level of evidence.

The limitations of this study and the novelty of its findings delineate a clear and high-impact agenda for future research. The immediate next step is a large, prospective, multi-center observational study to validate that post-procedural glycemia, infection, and cardiac events are independent predictors of mortality after adjusting for MELD, CTP, ACLF Grade, and renal disease stage. Furthermore, the most actionable finding (post-procedural glycemia) provides a compelling rationale for a high-impact randomized controlled trial on aggressive versus standard peri-procedural glycemic control. Given the catastrophic mortality associated with infection, another RCT is needed to test enhanced prophylactic strategies, such as extended antibiotic regimens. Similarly, the 85.7% mortality from cardiac events demands prospective studies on advanced cardiac risk stratification beyond standard echocardiography, such as stress echocardiography or right-heart catheterization. The goal should be the development of a new prognostic model that incorporates these newly identified, potent risk factors—glycemic status, infection risk, and cardiac function, as the data confirms that a MELD score alone is insufficient for this population. Such a tool would provide clinicians with a far more accurate, individualized 90-day mortality risk stratification to guide the critical decision of whether to proceed with a TIPSS in these high-risk patients.

## 5. Conclusions

In this retrospective single-center study of 68 cirrhosis patients with moderate-to-severe pre-existing kidney disease undergoing a TIPSS, overall mortality was 36.8% at mean follow-up of 6.7 months. Traditional severity scores (MELD, CTP) did not predict survival. Exploratory analyses identified associations between mortality and three potentially modifiable factors: uncontrolled diabetes (HbA1c ≥ 7.5%) pre-TIPSS, post-TIPSS infection, and cardiac events. These hypothesis-generating findings require prospective validation before clinical implementation. Until confirmatory studies are available, patient selection for a TIPSS in the setting of PKD should continue to rely on established criteria while recognizing the substantial mortality risk in this population.

## Figures and Tables

**Table 1:** Baseline clinical and laboratory characteristics.

Parameter	Mean ± SD	Median (IQR)	Range
**Demographics**			
Age (years)	61.0 ± 8.3	60.0 (58.0–63.6)	45–78
**Laboratory Parameters**			
Hemoglobin (g/dL)	9.1 ± 1.5	9.0 (8.4–9.3)	6.3–12.8
Total leukocyte count (×10^3^/μL) *	6.2	6.0 (5.2–7.1)	2.0–19.6
Platelet count (×10^3^/μL) *	116.8	120.0 (102.2–125.0)	57–331
Total bilirubin (mg/dL)	1.8 ± 1.0	1.7 (1.3–2.0)	0.2–5.1
Direct bilirubin (mg/dL) *	0.6	0.8 (0.5–0.9)	0.1–2.7
AST (IU/L) *	47.2	45.0 (39.4–52.1)	11–533
ALT (IU/L) *	28.2	27.5 (23.0–31.0)	11–327
Alkaline phosphatase (IU/L) *	120.4	116.0 (104.9–129.0)	47–446
Albumin (g/dL)	2.9 ± 0.6	2.9 (2.7–3.1)	1.8–4.2
INR *	1.6	1.5 (1.4–1.6)	1.2–5.5
Blood urea (mg/dL) *	63.0	65.3 (51.3–75.1)	16.6–238.0
Serum creatinine (mg/dL) *	1.9	1.7 (1.6–1.8)	1.5–5.1
Serum sodium (mEq/L)	133.5 ± 6.4	134.0 (132.0–136.0)	115–147
Serum potassium (mEq/L) *	4.3	4.2 (4.0–4.4)	3.0–11.0
**Severity Scores**			
Child–Turcotte–Pugh score	8.4 ± 1.5	8.5 (8.0–9.0)	5–12
MELD 3.0 score	22.4 ± 5.8	22.8 (21.0–24.0)	10.2–35.4
CLIF-C AD score	59.4 ± 8.6	59.0 (57.0–62.0)	44–85
CLIF-C organ failure score *	6.7	7.0 (6.0–7.0)	5–10
eGFR (mL/min/1.73m^2^)	38.5 ± 11.2	41.5 (40.0–43.6)	12–55

* Geometric mean reported for non-normally distributed variables. AST—aspartate transaminase, ALT—alanine transaminase, INR—international normalized ratio, MELD—model for end-stage liver disease, CLIF-C—chronic liver failure consortium, AD—acute decompensation score.

**Table 2:** Changes in laboratory parameters following TIPSS placement.

Parameter	Pre-TIPSS	Post-TIPSS	Geometric Mean Ratio (95% CI)	*p*-Value
**Hepatic Function**				
Total bilirubin (mg/dL) *	1.48	2.28	1.54 (1.36–1.75)	<0.001
Direct bilirubin (mg/dL) *	0.63	1.00	1.57 (1.38–1.79)	<0.001
Indirect bilirubin (mg/dL) *	0.78	1.18	1.51 (1.30–1.75)	<0.001
AST (IU/L) *	47.2	117.9	2.50 (1.88–3.32)	<0.001
ALT (IU/L) *	28.2	64.1	2.27 (1.73–2.97)	<0.001
Albumin (g/dL) *	2.89	2.74	0.95 (0.91–0.99)	0.017
INR *	1.56	1.82	1.17 (1.12–1.22)	<0.001
**Renal Function**				
Serum creatinine (mg/dL) *	1.93	1.75	0.91 (0.83–0.99)	0.031
**Hematological**				
Total leukocyte count (×10^3^/μL) *	6.20	8.18	1.32 (1.18–1.48)	<0.001
**Electrolytes**				
Serum sodium (mEq/L) *	133.4	136.5	1.02 (1.01–1.04)	0.004
**Severity Scores**				
CTP score *	8.27	8.74	1.06 (1.01–1.10)	0.016
MELD 3.0 score *	21.6	21.8	1.01 (0.95–1.07)	0.740

* Geometric mean reported for non-normally distributed variables. AST—aspartate transaminase, ALT—alanine transaminase, INR—international normalized ratio, MELD—model for end-stage liver disease.

**Table 3:** Causes of readmission within 30 days and causes of death during follow-up.

Reason for Readmission		N (%)	
Hepatic encephalopathy		5 (35.7%)	
Sepsis		3 (21.4%)	
Symptomatic ascites		2 (14.3%)	
Gastrointestinal bleeding		1 (7.1%)	
Non-GI bleeding		1 (7.1%)	
Dyselectrolytemia		1 (7.1%)	
Progressive liver failure		1 (7.1%)	
**Cause of Death**	**In-Hospital** **(n = 8)**	**Post-Discharge (n = 17)**	**Total (n = 25)**
**Primary Causes**			
Sepsis/Infection	4 (50.0%)	7 (41.2%)	11 (44.0%)
Progressive liver failure	3 (37.5%)	5 (29.4%)	8 (32.0%)
Gastrointestinal bleeding	0 (0%)	3 (17.6%)	3 (12.0%)
Cardiac failure	1 (12.5%)	1 (5.9%)	2 (8.0%)
Renal failure	0 (0%)	1 (5.9%)	1 (4.0%)

**Table 4:** Major predictors of overall mortality.

Factor	Deaths/Total (%)	Relative Risk (95% CI)	Odds Ratio (95% CI)	*p*-Value
**Diabetes Control**				
Controlled	7/27 (25.9%)	Reference	Reference	
Uncontrolled	16/29 (55.2%)	2.13 (1.04–4.36)	3.52 (1.14–10.88)	0.028
**Post-Procedural Glycemic Status**				
Controlled	6/29 (20.7%)	Reference	Reference	
Uncontrolled	19/39 (48.7%)	2.35 (1.08–5.15)	3.64 (1.22–10.90)	0.019
**Post-Procedural Infections**				
No	18/58 (31.0%)	Reference	Reference	
Yes	7/10 (70.0%)	2.26 (1.26–4.04)	5.11 (1.18–22.16)	0.019
**Post-TIPSS Cardiac Events**				
No	19/61 (31.1%)	Reference	Reference	
Yes	6/7 (85.7%)	2.75 (1.67–4.54)	14.21 (1.60–126.30)	0.005

## Data Availability

The data supporting the findings of this study are available from the corresponding author upon reasonable request.

## Abbreviations

ACLF	Acute-on-Chronic Liver Failure
ALD	Alcohol-Related Liver Disease
ALT	Alanine Aminotransferase
AST	Aspartate Aminotransferase
AVB	Acute Variceal Bleeding
CI	Confidence Interval
PKD-EPI	Chronic Kidney Disease Epidemiology Collaboration
CLIF-C AD	Chronic Liver Failure Consortium Acute Decompensation
CTP	Child–Turcotte–Pugh
HE	Hepatic Encephalopathy
HRS	Hepatorenal Syndrome
ICU	Intensive Care Unit
MASH	Metabolic Dysfunction-Associated Steatohepatitis
MELD	Model for End-Stage Liver Disease
PKD	Pre-existing Kidney Disease
RA	Refractory Ascites
TIPSS	Transjugular Intrahepatic Portosystemic Shunt
